# The Requirements of Partial Anæsthesia

**Published:** 1907-06-22

**Authors:** J. Aikman

**Affiliations:** Guernsey


### Points in Surgery.

### THE REQUIREMENTS OF PARTIAL ANÆSTHESIA.

By J. AIKMAN, M.D.Glasg., Guernsey.

### II. IN RELIEF OF PAIN.

It must be admitted that anæsthesia by ether is a long, deep, and effectual intoxication. It has two drawbacks: (1) the effect of the large dose upon the respiratory apparatus; (2) the difficulty of suiting the anæsthesia to conditions in which the pain is intermittent. Simpson's choice of chloroform meets the latter requirement, especially in obstetric practice; it does not damage the lungs, but when rapidly administered it is dangerous to the heart. Investigation showed that danger arises from a too great concentration of the vapour of this drug. In non-operative obstetric cases partial anæsthesia need seldom be exceeded. The comparative safety of such an anæsthesia—and the risk is little more than that of travelling by rail—created the cry for its use in many minor degrees of pain. Nitrous oxide gas passed from the realm of a laboratory experiment into medical and dental practice; but its usefulness is limited—ethyl chloride and its congeners hold the favour of the day. The potent and rapid action of ethyl-chloride requires specially constructed inhalers, as much for the safety of the administrator as of the patient, and although it enters as freely into the blood discs as chloroform itself, and even more markedly affects the physical characters of the blood, the heart muscle is only paralysed by about 19 times the proportionate amount. In these ways it meets the demands of the public; it is safe in skilled hands, provided only that the patient does not neglect the needful preparation.

It was a great evolution which led Liebreich to introduce chloral hydrate as an agent which produces chloroform slowly in the blood, brings sleep, lessens reflexes, and lowers the temperature in fever. It is true that his hypothesis was discredited by after-observers, but the drug marked an epoch in the control of distressing symptoms to which opium and its alkaloids were inapplicable. The headache and the weary sleeplessness of the early stage of the continued fevers make a bad precedent for the difficulties to be met and conquered in the later stages of the disease. Moderate doses of chloral hydrate secure some hours of rest, in the days before toxic delirium destroys the consciousness of the sufferer. He meets his later stages better equipped with strength for the fight towards recovery. The myocardium suffers little, if at all, provided that the drug is withheld in due season. It is little wonder that we gave to chloral the reception which we give to the morning sunshine—we ruled our day by it—and it came to a bad end by abuse. In the early stages of fever, in sleeplessness from pain which is not paroxysmal in character, and which is moderate in amount; in the stress of sorrow or anxiety, to which it is desired to give a temporary rest, there is no better drug than chloral hydrate with either bromide of potassium or cannabis indica. No cardiac tonic need be added unless it is desired to press the drug on more than three consecutive nights—which should seldom be the case.

Sulphonal inaugurated a new series of hypnotic agents, and some new developments. It acted rapidly if dissolved; more slowly, and frequently over a second night, if left to the solvent agencies of the digestive juices. But in the latter case the intervening day was abnormal—the patient was either stupid, forgetful, and heavy, or he was stimulated and excited, in proportion to the dose. It was misused in either event, and it seemed to me that deterioration of the blood corpuscles and hæmatoporphyrinuria followed more frequently when the patient continued small doses and kept up much physical exertion, and in cases in which the larger and less frequent dose failed to control delirium in which muscular exertion was a prevailing feature. I have seen no fatal case, but some in which there was cause for great anxiety. It has always appeared to me to be a drug in which an after-rest was imperative.

Tetronal and trional obviated in some degree the effects on the blood corpuscles, and were more rapid hypnotics, but they failed to gain an equal recognition in the treatment of disease conditions. Veronal is, of all the hypnotics, the most of a surprise. Skilfully used, the patient rests from business and worry, and begins life again with a promise of natural sleep. But, like most surprises, it loses its character by repetition—it should be given, rather than prescribed. I saw one fatal case, in which half an ounce had been taken. In it the facial reflexes were abolished except the vasomotor dilatation under the scratching of the finger nail; while the adjoining portions of the neck supplied by the spinal nerves still responded. The association interested me, as the drug had always seemed to me to be the most useful in the accumulations of mental worry which required only rest for their re-arrangement and cure.

Of these many inventions there will be no end; and some will serve well the purpose of their day; but it behoves the practitioner to remember that natural sleep is the heritage of man, that it is in his power to conserve it for him, and that he is responsible for guarding him against the misuse of such drugs as may rob him of the sweet sleep which was his possession before the evil times of yesterday.

---

Electro-medical Apparatus.—Messrs. Krupka and Jacoby, of 61 and 62 Watling Street, E.C., contractors to H. M. Government, issue an illustrated price-list of electrical apparatus in use in every branch of modern electro-therapeutics. The list is extremely useful for reference purposes, and the price of every piece of apparatus is clearly given.

